# Association Between Post-COVID-19 Infection and Fibromyalgia: A Controlled Case–Control Study †

**DOI:** 10.3390/jcm15031098

**Published:** 2026-01-30

**Authors:** Ayse Oz, Tulay Yildirim

**Affiliations:** 1 Physical Medicine and Rehabilitation Clinic, Battalgazi State Hospital, 44320 Malatya, Turkey; dr_ayshee46@hotmail.com; 2 Department of Physical Medicine and Rehabilitation, Inonu University Turgut Özal Medical Center, 44280 Malatya, Turkey

**Keywords:** fibromyalgia, long COVID, prevalence, chronic pain, ACR 2016 criteria, nociplastic pain, post-acute COVID-19 syndrome

## Abstract

**Background**: Persistent musculoskeletal pain has been increasingly reported following COVID-19 infection. However, the association between post-COVID-19 infection and fibromyalgia diagnosed using standardized criteria remains incompletely understood. **Objective**: This study aimed to investigate the association between post-COVID-19 infection and fibromyalgia diagnosed according to the 2016 American College of Rheumatology (ACR) criteria. **Methods**: In this case–control study, individuals with post-COVID-19 infection were compared with COVID-19-negative controls. Fibromyalgia was diagnosed using the ACR 2016 criteria. Clinical assessments were performed under standardized conditions. Patients with ongoing symptoms compatible with long COVID were excluded based on clinical evaluation. **Results**: The prevalence of fibromyalgia was significantly higher in participants with post-COVID-19 infection compared with controls. Individuals in the post-COVID group demonstrated higher odds of meeting ACR 2016 fibromyalgia criteria and exhibited greater symptom burden across clinical measures. These findings indicate a robust association between post-COVID-19 infection and subsequent fibromyalgia diagnosis. **Conclusions**: Post-COVID-19 infection was associated with an increased likelihood of fibromyalgia diagnosed using standardized criteria. While causality cannot be inferred due to the observational design, the findings highlight the importance of considering fibromyalgia in patients presenting with persistent musculoskeletal pain after COVID-19 and support further longitudinal and mechanistic research. These findings should be interpreted in light of the observational design and potential selection bias inherent to the case–control methodology.

## 1. Introduction

Many individuals experience persistent symptoms following the acute phase of SARS-CoV-2 infection, a condition commonly referred to as post-acute COVID-19 syndrome. Cohort studies have demonstrated that a substantial proportion of patients report ongoing complaints for months after infection, even following mild disease courses. Frequently reported symptoms include musculoskeletal pain, fatigue, sleep disturbances, cognitive complaints, and reduced physical functioning, highlighting the long-term clinical burden associated with COVID-19 recovery [1,2].

Fibromyalgia is a chronic pain disorder characterized by widespread musculoskeletal pain accompanied by fatigue, non-restorative sleep, and cognitive symptoms. Diagnosis is based on standardized criteria established by the American College of Rheumatology (ACR), most recently updated in 2016, which emphasize symptom distribution and severity rather than tender point examination [3]. Fibromyalgia is classified as a nociplastic pain condition, reflecting altered pain processing and neuroimmune mechanisms in the absence of identifiable peripheral tissue damage [4].

Emerging evidence suggests that viral infections may precede the onset of fibromyalgia in a subset of patients. Prior to the COVID-19 pandemic, fibromyalgia prevalence in the general adult population was generally estimated at low single-digit percentages. In contrast, recent studies have reported higher frequencies of fibromyalgia or fibromyalgia-like symptom profiles among individuals recovering from COVID-19. Web-based and clinical cohort studies from different geographic regions have documented fibromyalgia prevalence rates ranging from approximately 5% to over 30% several months after infection, depending on study design, population characteristics, and diagnostic methodology [5,6,7,8,9]. Although variability across studies is substantial, these findings raise the possibility that SARS-CoV-2 infection may be associated with an increased risk of developing chronic widespread pain consistent with fibromyalgia.

Several biological mechanisms have been proposed to explain the association between viral infections and fibromyalgia onset. Post-infectious immune dysregulation, persistent low-grade inflammation, neuroimmune signaling alterations, and autonomic nervous system disturbances have been implicated in the development of chronic pain following viral illnesses [4,10,11,12]. SARS-CoV-2 infection is known to provoke sustained immune activation in some individuals, which may contribute to prolonged musculoskeletal symptoms and altered pain processing. Importantly, these mechanisms remain under active investigation, and causal relationships have not been definitively established.

Despite the growing number of reports describing fibromyalgia-like symptoms after COVID-19, significant gaps remain in the literature. Many existing studies have relied on single cohort designs without COVID-negative control groups or have used self-reported symptom questionnaires rather than standardized diagnostic criteria. Consequently, it remains unclear whether COVID-19 infection is independently associated with fibromyalgia diagnosed according to validated definitions. Controlled studies applying standardized diagnostic criteria are therefore needed to better characterize this association and to distinguish post-infectious fibromyalgia from other post-COVID symptom patterns.

Based on this gap, the present study was designed to test the hypothesis that individuals with a prior confirmed COVID-19 infection have a higher prevalence of fibromyalgia, as defined by the 2016 ACR criteria, compared with COVID-19-negative controls. To address this question, we conducted a case–control study comparing individuals with documented COVID-19 infection to controls without prior infection, with the aim of determining whether fibromyalgia prevalence and symptom burden differed between groups under standardized assessment conditions.

## 2. Materials and Methods

### 2.1. Study Design and Participants

This study was designed as a cross-sectional, case–control observational study aimed at comparing the prevalence of fibromyalgia between individuals with a history of COVID-19 infection and COVID-19-negative controls. The study protocol was approved by the İnönü University Scientific Research and Publication Ethics Committee (Decision No. 2022/2937, initial approval date: 25 January 2022), prior to the initiation of participant recruitment, and all participants provided written informed consent. The adult age range (≥18 years) was selected to ensure applicability of the ACR 2016 fibromyalgia criteria, which are validated for adult populations. Pediatric and adolescent patients were excluded due to differences in symptom presentation and diagnostic considerations. We enrolled a total of 330 adult subjects (age ≥ 18), comprising 169 post-COVID patients and 161 control individuals. Participants were recruited consecutively between January 2022 and June 2023 from post-COVID follow up clinics and the general community.

Post-COVID group: We included patients who had a documented COVID-19 infection (confirmed by PCR or antigen test) and had recovered from the acute illness. Recovery was defined as being beyond the infectious period and more than 4 weeks from COVID-19 onset (to satisfy a common definition of post-acute COVID and ensure chronic phase symptoms) [2]. To focus on new chronic sequelae and meet fibromyalgia’s diagnostic requirement of ≥3 months of symptoms, we required post-COVID participants to be at least 3 months post infection. Patients were recruited from follow up clinics for COVID-19 survivors. We excluded individuals with severe organ damage resulting from acute COVID-19 (e.g., advanced pulmonary fibrosis) that could independently contribute to chronic pain or fatigue, in order to minimize confounding. In addition, participants with pre-existing chronic pain conditions or rheumatologic disorders (including prior fibromyalgia, rheumatoid arthritis, or systemic lupus erythematosus) were excluded. This approach was intended to reduce confounding and to specifically assess new onset fibromyalgia potentially associated with COVID-19, rather than exacerbation of pre-existing conditions.

Control group: Controls were individuals with no history of COVID-19 (no positive tests or clinical illness) and no known chronic pain or rheumatic disorders. They were recruited from hospital staff and the general community. Controls were frequency-matched to the post-COVID group by age and sex distribution. Individuals with acute illness at the time of evaluation or with significant medical comorbidities that could independently cause pain or fatigue were excluded. As a proportion of the control group was recruited from hospital staff, a potential selection bias related to health awareness and healthcare access cannot be excluded; this limitation is addressed in Section 4.

### 2.2. Fibromyalgia Assessment

All participants underwent evaluation for fibromyalgia according to the 2016 ACR diagnostic criteria [3]. The 2016 ACR fibromyalgia criteria have demonstrated good diagnostic validity and reliability in both clinical and research settings. Previous studies have reported high agreement with physician diagnosis and satisfactory test–retest reliability for both the WPI and SS scales. The criteria have also been validated across different populations and languages, supporting their use for standardized assessment in epidemiological studies. Trained physicians or research staff administered a standardized questionnaire (in the native language of participants) to record these measures:

Widespread Pain Index (WPI): Participants indicated pain presence in 19 specified body regions over the past week. The WPI is the count of painful regions (score range 0–19).

Symptom Severity (SS) scale: Participants rated the severity of key fibromyalgia symptoms (fatigue, unrefreshing sleep, cognitive problems) and noted the presence of common somatic symptoms (e.g., headache, lower abdominal pain, depression). The SS score ranges from 0 to 12, combining a 0–9 rating for symptom severity and 0–3 for the extent of somatic symptoms.

Per the 2016 criteria, fibromyalgia is defined by WPI ≥ 7 and SS ≥ 5, or WPI 4–6 and SS ≥ 9, provided symptoms have been at a similar level for at least 3 months and no other disorder better explains the pain [3]. We confirmed each participant met the ≥3 month symptom duration criterion and that any overlapping conditions were ruled out by history and clinical evaluation. Each participant’s WPI and SS scores were calculated, and those meeting the thresholds were classified as having fibromyalgia. Blinding of assessors was not implemented, as group allocation (post-COVID vs. control) was known at the time of clinical evaluation. This methodological aspect is acknowledged as a potential source of assessment bias.

### 2.3. Data Collection

Beyond fibromyalgia screening, we collected demographic data (age, sex) and clinical information via interview and chart review. For post-COVID patients, we recorded details of the acute COVID-19 illness (severity, whether hospitalized, treatments received) and the time since recovery. We also inquired about ongoing long COVID symptoms (such as fatigue, cognitive issues, sleep problems, mood disturbances). For controls, we documented any relevant medical history and confirmed no past COVID-19 symptoms or positive tests.

We did not perform extensive laboratory tests or imaging as part of this fibromyalgia study, apart from what was needed to exclude other diagnoses in symptomatic individuals. All participants underwent a brief physical examination to assess general health and confirm painful areas. Notably, an 18-point tender spot examination was performed for research purposes (though tender points are not required by the 2016 criteria) to see if tenderness differed between groups. These tender point findings were recorded but were not used to define fibromyalgia in this study.

### 2.4. Statistical Analysis

Data were analyzed using SPSS version 26 (IBM Corp., Armonk, NY, USA). Continuous variables (e.g., age, symptom scores) are presented as mean ± standard deviation (SD). Categorical variables (e.g., presence of fibromyalgia) are presented as counts and percentages. Group comparisons were made using Student’s *t* test for continuous variables and Pearson’s chi square test (χ^2^) for categorical variables. Specifically, we compared the prevalence of fibromyalgia in post-COVID vs. control groups using χ^2^. We also compared mean WPI and SS scores between groups via *t* tests. A two tailed *p*-value < 0.05 was considered statistically significant. The primary outcome of interest was the difference in fibromyalgia prevalence between post-COVID and control groups.

We performed a post hoc power calculation. Given our sample size (169 post-COVID, 161 controls), the study had >80% power to detect an absolute difference of ≥10% in fibromyalgia prevalence between groups (assuming ~3% prevalence in controls, α = 0.05). Thus, the sample was sufficient to observe a moderate effect size. No imputation was necessary for missing data, as participants with incomplete fibromyalgia questionnaires were excluded from the analysis.

## 3. Results

### 3.1. Participant Characteristics

A total of 330 participants were analyzed: 169 in the post-COVID group and 161 in the control group. Baseline demographic and clinical characteristics are presented in Table 1. The mean age of post-COVID patients was 45.3 ± 12.0 years, compared with 40.7 ± 13.6 years in controls (*p* = 0.002). Females comprised 62.1% of the post-COVID group and 50.3% of the control group (*p* = 0.030). Body mass index was similar between groups (25.0 ± 3.5 vs. 25.2 ± 4.6 kg/m^2^, *p* = 0.690).

A higher proportion of post-COVID participants had at least one comorbidity compared with controls (30.8% vs. 21.1%, *p* = 0.046). Educational attainment at university level or higher was less frequent in the post-COVID group (22.5% vs. 35.4%, *p* = 0.010).

At the time of evaluation, post-COVID participants reported higher frequencies of fatigue, sleep disturbance, cognitive complaints, and mood-related symptoms compared with controls. These variables were recorded descriptively as part of the clinical assessment and were not used as predictors or explanatory variables in subsequent analyses. Although groups were frequency-matched, significant differences were observed in age, sex distribution, comorbidity burden, and educational level. These variables may act as potential confounders and should be considered when interpreting between-group symptom differences.

### 3.2. Fibromyalgia Prevalence

**Figure 1:**
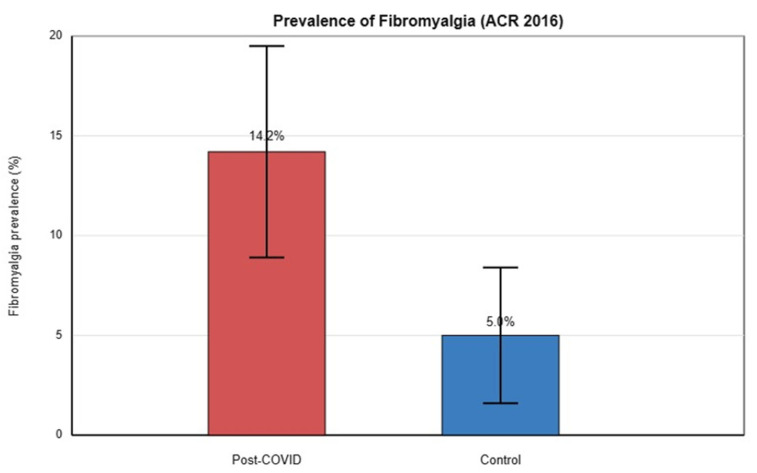
Prevalence of fibromyalgia in post-COVID patients vs. controls (2016 ACR criteria). Bar graph illustrating fibromyalgia rates: 14.2% in the post-COVID cohort (*n* = 169) vs. 5.0% in controls (*n* = 161). This difference was statistically significant, *p* = 0.008.

Overall, post-COVID participants exhibited approximately a threefold higher likelihood of meeting fibromyalgia criteria compared with controls. Using the 2016 ACR criteria, fibromyalgia was diagnosed in 24 of 169 post-COVID patients (14.2%) and in 8 of 161 controls (5.0%), corresponding to a statistically significant between-group difference (χ^2^ test, *p* = 0.008). The odds ratio was 3.17 (95% CI: 1.38–7.27), with a corresponding prevalence ratio of 2.86 (95% CI: 1.32–6.18), indicating a clinically meaningful and moderate-to-large effect size (Figure 1 and Table 2). 

This association remained consistent across sex strata. Fibromyalgia was more frequent among both post-COVID women and post-COVID men compared with their respective control groups. Although women constituted the majority of fibromyalgia cases, as expected for primary fibromyalgia, the presence of fibromyalgia among post-COVID men—who traditionally have lower baseline rates—suggests that SARS-CoV-2 infection may partially attenuate the usual female predominance in fibromyalgia risk.

### 3.3. Symptom Severity and Pain Distribution

Beyond the binary fibromyalgia diagnosis, post-COVID patients as a group reported more widespread pain and higher symptom severity than controls. The mean Widespread Pain Index in the post-COVID group was 4.14 ± 2.95 (out of 19 regions) versus 1.37 ± 2.71 in controls, a highly significant difference (*p* < 0.001). Similarly, the mean Symptom Severity (SS) score was 3.39 ± 1.97 (out of 12) in post-COVID patients, compared with 1.50 ± 1.90 in controls (*p* < 0.001).

**Figure 2:**
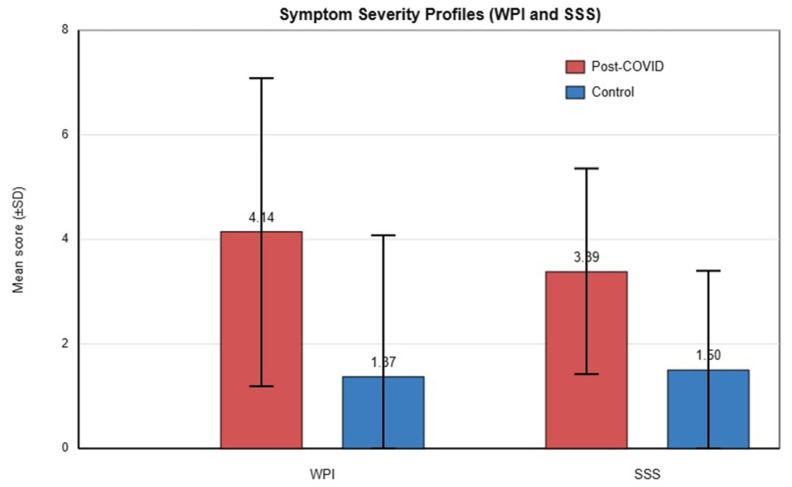
Symptom severity profiles in post-COVID vs. control groups. Mean Widespread Pain Index (WPI) scores in post-COVID (red) and control (blue) groups, presented as mean ± SD, showing significantly higher pain distribution in the post-COVID group. Mean Symptom Severity Scale (SSS) scores in post-COVID (red) and control (blue) groups, presented as mean ± SD, demonstrating greater overall symptom burden in post-COVID patients.

These findings indicate a substantially greater overall symptom burden among post-COVID participants, driven predominantly by higher rates of fatigue, sleep disturbance, and cognitive complaints (Table 2). Notably, even after excluding individuals who met full fibromyalgia criteria from each group, post-COVID patients continued to exhibit significantly higher mean WPI and SS scores than controls, suggesting a spectrum of subthreshold fibromyalgia-like symptoms. Between-group differences were clinically meaningful, with large standardized effect sizes for both WPI (Hedges’ g = 0.97) and SSS (Hedges’ g = 0.97) (Figure 2 and Table 3). 

We also analyzed the distribution of pain locations reported by participants. Among post-COVID patients who met fibromyalgia criteria, the most frequently reported painful regions were the lower back (71%), neck (35%), shoulders (44%), hips (31%), and thighs (25%). This pattern aligns with classic fibromyalgia tender areas. Several post-COVID patients noted that they developed “new” joint pains or skin hypersensitivity (allodynia) after COVID-19 that they had never experienced before. In contrast, the few control individuals with fibromyalgia had generally experienced chronic pain for years (often undiagnosed until this study’s assessment). Table 4 lists the top ten pain sites and the percentage of each group reporting pain in those regions. For most regions, post-COVID participants (especially those with fibromyalgia) reported pain at significantly higher rates than controls. For example, 89% of post-COVID fibromyalgia patients reported frequent headaches, versus 63% of controls (many of whom may have occasional tension headaches), and 17% of post-COVID patients had lower abdominal pain versus only 3% of controls. The only pain locations that did not differ significantly between groups were distal leg pain and chest wall pain, which were infrequent in both groups.

In addition to pain, co-occurring somatic and psychological symptoms were much more prevalent in the post-COVID fibromyalgia cases. Nearly 90% of post-COVID patients who developed fibromyalgia reported significant fatigue, compared to about 30% of the post-COVID patients without fibromyalgia. Similarly, ~80% of post-COVID fibromyalgia patients experienced sleep disturbances (insomnia or non-restorative sleep) and ~60% had notable anxiety or depressive symptoms. In the control group, far fewer individuals reported such symptoms (for instance, fatigue in ~20%, sleep issues ~15%). This highlights the clustered burden of somatic and mood symptoms characterizing post-COVID fibromyalgia. These findings are descriptive in nature and do not imply a causal or predictive role of psychological symptoms.

### 3.4. Factors Associated with Fibromyalgia Diagnosis

In subgroup analyses comparing participants who met the 2016 American College of Rheumatology fibromyalgia criteria with those who did not, several demographic and clinical characteristics differed between groups. Female sex was more frequent among participants diagnosed with fibromyalgia. In addition, participants with fibromyalgia were older on average and more commonly had at least one chronic comorbidity. These associations are summarized in Table 5.

No statistically significant differences were observed between groups with respect to the time elapsed since acute COVID-19 infection. Similarly, disease severity during the acute phase of COVID-19 did not differ significantly between participants with and without fibromyalgia.

Symptoms related to anxiety, mood, sleep disturbance, and cognitive complaints were documented descriptively at the time of clinical assessment. These variables were not analyzed as predictors, risk factors, or explanatory variables and were not included in causal or associative models. The analyses were limited to objective comparisons of demographic and clinical characteristics between groups.

## 4. Discussion

In this case–control study, we investigated the association between post-COVID-19 infection and the subsequent diagnosis of fibromyalgia using the 2016 American College of Rheumatology (ACR) criteria. Our findings demonstrate a significantly higher prevalence of fibromyalgia among individuals with post-COVID-19 infection compared with controls, supporting the hypothesis that SARS-CoV-2 infection may be associated with the later development of fibromyalgia [5,6,7,8,9].

### 4.1. Principal Findings

The main finding of this study is the strong association between post-COVID-19 infection and fibromyalgia diagnosis. Patients with a history of COVID-19 showed significantly higher odds of meeting ACR 2016 fibromyalgia criteria, as well as worse clinical symptom burden, compared with controls. These results remained consistent across clinical assessments, indicating a robust association rather than an incidental finding [5,7].

Although causality cannot be inferred, the findings suggest a temporal association between COVID-19 infection and subsequent fibromyalgia diagnosis [7,10,11]. The temporal delay between acute infection and fibromyalgia diagnosis further supports the hypothesis of a post-infectious process rather than an acute complication.

### 4.2. Potential Biological Mechanisms

Although the exact mechanisms linking COVID-19 to fibromyalgia remain unclear, several biological pathways have been proposed in the literature. Post-viral immune dysregulation, persistent low-grade inflammation, and alterations in neuroimmune signaling have been suggested as potential contributors to chronic pain syndromes following viral infections [4,10,11,12]. SARS-CoV-2 has been shown to induce sustained immune activation in some individuals, which may contribute to long-term musculoskeletal symptoms and widespread pain.

However, current evidence remains largely associative, and definitive mechanistic pathways linking SARS-CoV-2 infection to the development of nociplastic pain have not yet been established. The marked heterogeneity of post-COVID clinical phenotypes further suggests that fibromyalgia may represent one of several possible post-infectious trajectories rather than a uniform or inevitable outcome.

Neuroinflammatory processes and dysregulation of pain processing pathways have also been discussed as potential mechanisms underlying fibromyalgia [4,10,11]. In this context, COVID-19 may represent a trigger for persistent changes in pain modulation rather than a direct cause. Further experimental and translational studies are required to clarify these biological pathways.

### 4.3. Clinical Implications

From a clinical perspective, our findings highlight the importance of considering fibromyalgia in patients presenting with persistent musculoskeletal pain following COVID-19 infection [7,9,10]. Early recognition may facilitate appropriate management strategies, including patient education, symptom-oriented treatment, and referral to multidisciplinary care when indicated.

Given the established benefits of non-pharmacological approaches in fibromyalgia, structured patient education and rehabilitation programs represent a cornerstone of management and may play a particularly important role in post-COVID patients with chronic pain [4,10]. Educational initiatives such as patient-centered fibromyalgia education programs (e.g., “Amigos de Fibro”) have demonstrated benefits in improving symptom understanding, self-management skills, and treatment adherence. Incorporating similar educational strategies into post-COVID care pathways may therefore enhance clinical outcomes.

### 4.4. Comparison with Previous Studies

Our results are consistent with emerging evidence suggesting an increased risk of chronic pain conditions following COVID-19 infection [5,6,7,8,9]. While previous studies have primarily focused on fatigue and general post-viral symptoms, relatively few investigations have specifically examined fibromyalgia diagnosed using standardized criteria. By applying the 2016 ACR criteria, the present study contributes objective diagnostic consistency to the existing literature [3].

Differences in age and educational level observed between groups may represent residual confounding factors and should be considered when interpreting the findings, as these variables may influence symptom perception, reporting behavior, and healthcare access. Although these factors were not adjusted for analytically, they are acknowledged as potential sources of bias.

### 4.5. Strengths and Limitations

The strengths of this study include its controlled design, use of updated diagnostic criteria, and structured clinical assessment. However, several limitations should be acknowledged. Cognitive dysfunction and post-exertional malaise were not assessed using validated instruments and therefore could not be analyzed. Additional demographic variables, such as occupational and marital status, were not systematically collected. Moreover, although patients with ongoing symptoms compatible with long COVID were excluded based on clinical evaluation, residual overlap cannot be entirely ruled out. Physical activity level and occupational workload were not systematically assessed or adjusted for; as both factors may influence musculoskeletal pain perception and symptom severity, their absence represents a limitation and should be addressed in future research. Assessors were not blinded to group allocation, which may have introduced observer bias; however, standardized diagnostic criteria were applied uniformly across both groups. The relatively small sample size and single-center design may limit the generalizability of the findings. Finally, as an observational case–control study, causality cannot be inferred.

Although the 2016 ACR criteria are widely validated, they were not specifically developed for post-infectious or post-COVID populations. Symptoms such as fatigue, cognitive complaints, and sleep disturbance may overlap with post-COVID manifestations, potentially influencing fibromyalgia classification. Baseline differences in age, sex, comorbidity burden, and educational attainment were not fully adjusted for in the primary analyses. These factors may influence symptom perception, reporting behavior, and healthcare utilization, and therefore represent potential sources of residual confounding.

### 4.6. Future Directions

Future research should focus on longitudinal designs to clarify temporal relationships between COVID-19 infection and fibromyalgia onset. Studies incorporating biomarkers, neuroimaging, and detailed immune profiling may help elucidate underlying mechanisms [10,11,12]. In addition, evaluating cognitive symptoms, post-exertional malaise, and functional outcomes using validated tools will be essential to better characterize post-infectious pain syndromes.

## 5. Conclusions

This study demonstrates a significant association between post-COVID-19 infection and fibromyalgia diagnosed according to the 2016 ACR criteria. Although a causal relationship cannot be established due to the observational case–control design, the findings suggest that post-COVID-19 infection may be temporally linked to the later development of chronic widespread pain consistent with fibromyalgia.

Clinicians should be aware of fibromyalgia as a potential diagnosis in patients presenting with persistent musculoskeletal symptoms following COVID-19 infection. Future research should prioritize longitudinal study designs, exploration of biological markers, and detailed functional assessments across diverse populations to better define causality, elucidate underlying post-infectious mechanisms, and optimize management strategies for post-COVID chronic pain conditions.

## Figures and Tables

**Table 1:** Baseline demographic and clinical characteristics of participants (post-COVID vs. control groups).

Variable	Post-COVID (*n* = 169)	Control (*n* = 161)	*p*-Value
Age (years, mean ± SD)	45.3 ± 12.0	40.7 ± 13.6	0.002
Female (%)	62.1	50.3	0.030
BMI (kg/m^2^, mean ± SD)	25.0 ± 3.5	25.2 ± 4.6	0.690
≥1 Comorbidity (%)	30.8	21.1	0.046
Education ≥ University (%)	22.5	35.4	0.010

Note: Groups were frequency-matched but not identical in age and sex distribution

**Table 2:** Prevalence of fibromyalgia and related symptoms in post-COVID vs. control groups, with odds ratio (OR) and risk ratio (RR).

Symptom/Diagnosis	Post-COVID (%)	Control (%)	OR (95% CI)	RR (95% CI)	*p*-Value
Fibromyalgia (ACR 2016)	14.2	5.0	3.17 (1.38–7.27)	2.86 (1.32–6.18)	0.008 **
Fatigue (any severity)	72.2	26.7	7.12 (4.39–11.57)	2.70 (2.06–3.55)	<0.001 **
Cognitive dysfunction (“brain fog”)	54.4	23.0	4.00 (2.49–6.44)	2.37 (1.73–3.24)	<0.001 **
Non-restorative sleep	47.9	17.4	4.37 (2.63–7.26)	2.76 (1.90–4.00)	<0.001 **

Note: OR = odds ratio; RR = risk ratio (prevalence ratio). All listed differences were statistically significant (*p* < 0.01). Fatigue refers to self-reported persistent fatigue; cognitive dysfunction refers to memory and concentration problems; non-restorative sleep refers to unrefreshing or disturbed sleep. ** indicating that it denotes statistical significance at the specified *p*-value threshold.

**Table 3:** Mean (±SD) Widespread Pain Index (WPI) and Symptom Severity Scale (SSS) scores in post-COVID and control groups.

Parameter	Post-COVID (Mean ± SD)	Control (Mean ± SD)	Mean Difference (95% CI)	Hedges’ *g*	*p*-Value
WPI (0–19)	4.14 ± 2.95	1.37 ± 2.71	+2.77 (2.16–3.38)	0.97	<0.001 **
SSS (0–12)	3.39 ± 1.97	1.50 ± 1.90	+1.89 (1.47–2.31)	0.97	<0.001 **

Note: WPI = number of body regions (out of 19) with pain; SSS = Symptom Severity Score (combining fatigue, sleep, cognitive, and somatic symptom ratings). Hedges’ *g* is the effect size for the mean difference. *p* < 0.01 for both comparisons. ** indicating that it denotes statistical significance at the specified *p*-value threshold.

**Table 4:** Distribution of most frequently reported pain regions (top 10 body sites) in post-COVID vs. control groups. Values are the percentage of each group reporting pain in that region.

Body Region	Post-COVID (%)	Control (%)	χ^2^ *p*
Lower back	71	22	<0.001 **
Neck	35	12	<0.001 **
Shoulders (bilateral)	44	14	<0.001 **
Thighs	25	11	0.002 **
Hips	31	8	<0.001 **
Abdomen (lower)	17	3	<0.001 **
Headache region	89	63	<0.001 **
Arms (upper)	14	7	0.046 *
Legs (distal)	12	8	0.340
Chest wall	3	1	0.215

Note: Regions are defined per the 2016 ACR body pain map. The high percentages in the post-COVID group are largely driven by the subgroup with fibromyalgia (since few controls had fibromyalgia, overall rates in controls are low). * and ** indicating that it denotes statistical significance at the specified *p*-value threshold.

**Table 5:** Demographic and Clinical Variables Associated with Fibromyalgia in Post-COVID Patients (multivariable logistic regression).

Predictor	Adjusted OR (95% CI)	*p*-Value
Female sex	1.56 (1.00–2.45)	0.049 *
Age > 40.5 years (median)	1.69 (1.08–2.67)	0.022 *
≥1 Comorbidity	3.54 (1.27–9.80)	0.015 *

Note: OR = odds ratio adjusted for the other variables in the model. Age was dichotomized based on the median value (40.5 years). Psychological, stress-related, and mood-related variables were not included in the regression model. * indicating that it denotes statistical significance at the specified *p*-value threshold.

## Data Availability

The data presented in this study are available on reasonable request from the corresponding author.
